# Supplementary material for: Defining the role of pulmonary endothelial cell heterogeneity in the response to acute lung injury
Source: eLife. 2020 Feb 24;9:e53072. doi: 10.7554/eLife.53072 (PMC7176435; doi:10.7554/eLife.53072)
Supplement: Transparent reporting form [file elife-53072-transrepform.pdf]

Westbrook Centre, Milton Road
Cambridge CB4 1YG
UK

P 01223 855340
W elifesciences.org
T @elife

eLife's transparent reporting form

We encourage authors to provide detailed information *within their submission* to facilitate the interpretation and replication of experiments. Authors can upload supporting documentation to indicate the use of appropriate reporting guidelines for health-related research (see EQUATOR Network), life science research (see the BioSharing Information Resource), or the ARRIVE guidelines for reporting work involving animal research. Where applicable, authors should refer to any relevant reporting standards documents in this form.

If you have any questions, please consult our Journal Policies and/or contact us: editorial@elifesciences.org.

Sample-size estimation

- You should state whether an appropriate sample size was computed when the study was being designed
- You should state the statistical method of sample size computation and any required assumptions
- If no explicit power analysis was used, you should describe how you decided what sample (replicate) size (number) to use

Please outline where this information can be found within the submission (e.g., sections or figure legends), or explain why this information doesn't apply to your submission:

Not applicable – no standard statistical procedures were applied to the single-cell RNA sequencing analysis in this study, and therefore no power analysis was performed during the study design.

Replicates

- You should report how often each experiment was performed
- You should include a definition of biological versus technical replication
- The data obtained should be provided and sufficient information should be provided to indicate the number of independent biological and/or technical replicates
- If you encountered any outliers, you should describe how these were handled
- Criteria for exclusion/inclusion of data should be clearly stated
- High-throughput sequence data should be uploaded before submission, with a private link for reviewers provided (these are available from both GEO and ArrayExpress)

Please outline where this information can be found within the submission (e.g., sections or figure legends), or explain why this information doesn't apply to your submission:

Replicate information for single-cell RNA sequencing of (1) lung endothelium, (2) adult mouse lung (control), and (3) adult mouse lung (H1N1 injury) can be found in the Materials and Methods section under "Single-cell RNA sequencing using In-Drop and the GemCode platform." Single-cell RNA sequencing data is available on the gene expression omnibus (GEO) with accession GEO:GSE128944. Replicate information for immunofluorescence and RNAscope analysis can be found in the legends for Figure 3, Figure 3 – Figure Supplements 1-2, and Figure 5 – Figure Supplements 1 and 5. Replicate information for quantification of EdU incorporation by flow cytometry can be found in the legend for Figure 5. Replicate information for qRT-PCR can be found in the legend for Figure 1 – Figure Supplement 3.

Statistical reporting

- Statistical analysis methods should be described and justified
- Raw data should be presented in figures whenever informative to do so (typically when N per group is less than 10)
- For each experiment, you should identify the statistical tests used, exact values of N, definitions of center, methods of multiple test correction, and dispersion and precision measures (e.g., mean, median, SD, SEM, confidence intervals; and, for the major substantive results, a measure of effect size (e.g., Pearson's r, Cohen's d)
- Report exact p-values wherever possible alongside the summary statistics and 95% confidence intervals. These should be reported for all key questions and not only when the p-value is less than 0.05.

Please outline where this information can be found within the submission (e.g., sections or figure legends), or explain why this information doesn't apply to your submission:

This study analyzes single-cell RNA sequencing data through clustering and dimension reduction to determine known and novel cell types, which were defined through expression of marker genes in each cluster. No standard statistical methods were applied to these datasets. Statistical analysis was performed on the following data: quantification of cell number in RNAscope and IHC experiments; quantification of expression in qRT-PCR experiments; and quantification of EdU incorporation by flow cytometry. In all of these cases, the statistical test(s) used are described in the methods section "Statistical analysis." P values are reported in the associated figure legends. Replicate information is also described in figure legends as indicated above.

(For large datasets, or papers with a very large number of statistical tests, you may upload a single table file with tests, Ns, etc., with reference to sections in the manuscript.)

Group allocation

- Indicate how samples were allocated into experimental groups (in the case of clinical studies, please specify allocation to treatment method); if randomization was used, please also state if restricted randomization was applied
- Indicate if masking was used during group allocation, data collection and/or data analysis

Please outline where this information can be found within the submission (e.g., sections or figure legends), or explain why this information doesn't apply to your submission:

Not applicable – this study used wild type C57BL/6J mice for all experiments and did not involve the assigning of patients or animal subjects into groups.

Additional data files ("source data")

- We encourage you to upload relevant additional data files, such as numerical data that are represented as a graph in a figure, or as a summary table
- Where provided, these should be in the most useful format, and they can be uploaded as "Source data" files linked to a main figure or table
- Include model definition files including the full list of parameters used
- Include code used for data analysis (e.g., R, MatLab)
- Avoid stating that data files are "available upon request"

Westbrook Centre, Milton Road
Cambridge CB4 1YG
UK
P 01223 855340
W elifesciences.org
T @elife

Please indicate the figures or tables for which source data files have been provided:

Single-cell RNA sequencing data is available on the gene expression omnibus (GEO) with accession GEO:GSE128944.

eLife Sciences Publications, Ltd is a limited liability non-profit non-stock corporation incorporated in the State of Delaware, USA, with company number 5030732, and is registered in the UK with company number FC030576 and branch number BR015634 at the address Westbrook Centre, Milton Road Cambridge CB4 1YG, UK | March 2019
